# Resistance to diet-induced adiposity in cannabinoid receptor-1 deficient mice is not due to impaired adipocyte function

**DOI:** 10.1186/1743-7075-8-93

**Published:** 2011-12-27

**Authors:** Maaike H Oosterveer, Anniek H Koolman, Pieter T de Boer, Trijnie Bos, Aycha Bleeker, Theo H van Dijk, Vincent W Bloks, Folkert Kuipers, Pieter JJ Sauer, Gertjan van Dijk

**Affiliations:** 1 Department of Pediatrics, University Medical Center Groningen; University of Groningen, P.O. Box 30.001 9700 RB Groningen, The Netherlands; 2 Department of Laboratory Medicine, Center for Liver Digestive and Metabolic Diseases, University Medical Center Groningen; University of Groningen, P.O. Box 30.001 9700 RB Groningen, The Netherlands; 3 Center for Behavior and Neurosciences, Unit Neuroendocrinology, University of Groningen, Nijenborgh 7, 9747 AG Groningen, The Netherlands

**Keywords:** CB_1_-receptor, diet-induced adiposity, fat tissue, lipogenesis, lipolysis

## Abstract

**Background:**

Overactivity and/or dysregulation of the endocannabinoid system (ECS) contribute to development of obesity. *In vitro *studies indicate a regulatory role for the cannabinoid receptor 1 (CB_1_) in adipocyte function and CB_1_-receptor deficient (*CB_1_^-/-^*) mice are resistant to high fat diet-induced obesity. Whether this phenotype of *CB_1_^-/- ^*mice is related to altered fat metabolism in adipose tissue is unknown.

**Methods:**

We evaluated adipose tissue differentiation/proliferation markers and quantified lipogenic and lipolytic activities in fat tissues of *CB_1_^-/- ^*and *CB_1_^+/+ ^*mice fed a high-fat (HF) or a high-fat/fish oil (HF/FO) diet as compared to animals receiving a low-fat chow diet. Comparison between HF diet and HF/FO diet allowed to investigate the influence of dietary fat quality on adipose tissue biology in relation to CB_1 _functioning.

**Results:**

The adiposity-resistant phenotype of the *CB_1_^-/- ^*mice was characterized by reduced fat mass and adipocyte size in HF and HF/FO-fed *CB_1_^-/- ^*mice in parallel to a significant increase in energy expenditure as compared to *CB_1_^+/+ ^*mice. The expression levels of adipocyte differentiation and proliferation markers were however maintained in these animals. Consistent with unaltered lipogenic gene expression, the fatty acid synthesis rates in adipose tissues from *CB_1_^-/- ^*and *CB_1_^+/+ ^*mice were unchanged. Whole-body and adipose-specific lipoprotein lipase (LPL) activities were also not altered in *CB_1_^-/- ^*mice.

**Conclusions:**

These findings indicate that protection against diet-induced adiposity in *CB_1_*-deficient mice is not related to changes in adipocyte function *per se*, but rather results from increased energy dissipation by oxidative and non-oxidative pathways.

## Background

The endocannabinoid system (ECS) comprises the endogenous cannabinoids (endocannabinoids or ECs), the cannabinoid receptors and the enzymes involved in the synthesis and degradation of endocannabinoids [1]. The two most studied ECs anandamide (AEA) and 2-arachidonoyl glycerol (2-AG) are amides and esters, respectively, of long-chain polyunsaturated fatty acids (PUFAs) [2]. To date, two G protein-coupled cannabinoid receptors have been identified. Because of its role in the central regulation of food intake and energy balance, the cannabinoid 1 (CB_1_)-receptor has emerged as an interesting drug target for treatment of obesity, dyslipidemia and insulin resistance. CB_2_-receptors, on the other hand, are mainly involved in immune function [3]. Administration of (endo)cannabinoids increases food intake, while CB_1_-receptor antagonism results in hypophagia [4]. Mice deficient for the cannabinoid 1 receptor gene (Cnr1, further announced as *CB_1_^-/- ^*mice) are lean and have less fat stores compared to their wild type littermates [5]. This reduction in adiposity appears to be independent of food intake [5], suggesting that *CB_1 _*deficiency alters the balance between energy intake, utilization and storage.

CB_1_-receptors are localized in the brain, peripheral nerves and several peripheral organs [3]. Adipose tissue represents an important peripheral node of the ECS and several *in vitro *studies indicate that CB_1_-receptor activity is critical for adipocyte function [5-7]. *CB_1 _*expression has been reported to increase during adipocyte maturation [8] and ECs are required for adipocyte differentiation and growth [6,9,10]. ECS signalling in adipocytes supposedly promotes lipid storage [6,11-13]*via *the induction of lipogenic genes and by increasing fatty acid uptake [5,6]. Accordingly, CB_1_-receptor antagonists arrest adipocyte proliferation and reduce lipogenic gene expression [6,13]. Since growth retardation in *CB_1_^-/- ^*mice is already apparent during the first weeks of life [5], appropriate CB_1_-receptor functioning may be critical for adipose tissue development *in vivo*.

Because EC receptor signalling impacts on energy balance, overactivity and/or dysregulation of the ECS have been proposed contribute to the development of obesity [7,11,14]. High-fat feeding induces obesity in rodents and has been reported to increase the EC tone in their adipose tissue [11,15] while *CB_1_*-deficient mice are resistant to high-fat diet-induced obesity [16]. Besides their effects on systemic energy expenditure, adipocyte-specific actions of the cannabinoid receptors potentially impact on adipose tissue physiology and thereby determine the degree of fat storage. Different types of dietary fats exert specific effects on body weight regulation [17] and n-3 PUFAs have been shown to decrease EC levels in adipocytes [18]. *In vitro *studies indicate that enhanced CB_1_-receptor signalling directs metabolism towards fat storage [6,11] while reducing fat oxidation [7,11,19]. In addition, it has been shown that peripheral CB_1_-receptor antagonism reduces the expression of lipogenic genes in mice fed a high-fat diet [20]. The protection against diet-induced obesity in *CB_1_*-deficient mice may therefore, at least in part, be related to changes in fat cell metabolism in these animals. Very few studies have however systematically evaluated the consequences of *CB_1 _*ablation for adipocyte biology *in vivo*. Furthermore, it is unknown whether CB_1_-receptor mediated changes in adipose tissue function contribute to high-fat diet induced obesity in rodents.

We therefore investigated whether resistance to diet-induced adiposity in *CB_1_*-deficient mice was related to functional changes in adipocyte biology. In order to evaluate the influence of dietary fat quality in relation to CB_1 _functioning in adipose tissue, we determined the expression levels of differentiation and proliferation markers, the rate of fatty acid synthesis and lipoprotein lipase (LPL) activity in epididymal fat pads of *CB_1_^+/+ ^*and *CB_1_*^-/- ^mice receiving a standard, low-fat chow diet, a high-fat (HF) or a HF diet in which part of the fat was replaced by fish oil (HF/FO diet). The results from this study indicate that obesity-resistance in *CB_1_-*deficient mice is not related to changes in adipocyte metabolism, but rather results from increased energy expenditure.

## Methods

### Animals and experimental design

Initial breeding pairs for the generation of cannabinoid 1 receptor gene (Cnr1, further announced as *CB_1_*) knockout mice were kindly provided by prof. dr. A. Zimmer, Laboratory of Molecular Neurobiology, Department of Psychiatry, University of Bonn, Germany. Male *CB_1_^+/+ ^*and *CB_1_*^-/- ^mice on a pure C57BL/6N background were bred within our own facility from heterozygous crossing. Animals were housed under light- and temperature-controlled conditions (lights on 4:00 AM-4:00 PM, 21°C) with free access to food and drinking water. From four weeks of age onwards, they were divided into groups and fed three different diets during six weeks. All diets were obtained from Abdiets, Woerden, The Netherlands. One group received normal laboratory chow (RMH-B), the second group received high-fat (HF; beef tallow) diet and the third group received a diet in which 42% (w/w) of the beef tallow was replaced by fish oil (HF/FO diet; menhaden oil). For diet composition see Additional File 1 Table S1. The HF/FO diet was renewed three times per week to prevent oxidation. Body weight and food intake were registered regularly. Separate cohorts of mice were generated to evaluate the effects on basal parameters, energy expenditure, lipogenic fluxes and lipolytic activities. Prior to all experiments, mice were subjected to a short postprandial fasting period of 3 hours (6-9 AM) with drinking water available to exclude acute postprandial effects without the induction of a fasting response. Experimental procedures were approved by the Ethics Committees for Animal Experiments of the University of Groningen.

### Determination of body composition by whole-body carcass analysis

During the 5^th ^week of dietary intervention, feces were collected over a 72-hour period. Fecal energy content was determined as previously described [21]. After six weeks of diet, animals were fasted (6-9 AM) and blood glucose concentrations were measured using a glucometer (Lifescan Benelux, Beerse, Belgium). Then, the mice were sacrificed by cardiac puncture under isoflurane anaesthesia and subcutaneous, epididymal, retroperitoneal and brown adipose fat depots were first collected and weighed to determine their mass. Intestine, organs, skin, and the remaining carcass including the head were separately collected to determine mesenteric, organ, non-removable subcutaneous and muscular fat mass, respectively. Therefore, these samples were dried until all moist was evaporated, after which the total (lean+fat) masses were determined. Lipids were subsequently extracted using Soxleth petroleum ether distillation. Then, the samples were again dried, after which the fat free masses were determined. The fat mass from the different samples was finally calculated by subtracting the lean mass from the total mass.

### Fat cell histology, basal plasma metabolite and gene expression analysis

Epididymal adipose tissue was quickly removed, snap-frozen in liquid nitrogen and stored at -80°C. Part of the fat tissue was fixed in 4% paraformaldehyde in PBS and embedded in paraffin. For adipocyte histology, 3 μm paraffin sections were stained with hematoxylin and eosin and analyzed at 10× magnification. The area of 240-420 fat cells per group was quantified using image analysis software (Qwin, Leica, Wetzlar, Germany). Fat cell area data were analyzed using the percent relative cumulative frequency (PCRF) approach and EC50 values were calculated according to Riachi *et al*. [22]. Adipose tissue-derived LPL activity was determined as described under '*lipolytic activity*'. Blood was centrifuged (4000 × g for 10 minutes at 4°C) and plasma was stored at -20°C. Plasma insulin, triglyceride, cholesterol and free fatty acids were quantified using commercially available kits (Mercodia, Uppsala, Sweden for insulin ELISA; Roche diagnostics, Mannheim, Germany for triglyceride and cholesterol; Wako Chemicals, Neuss, Germany for free fatty acids). Plasma leptin, resistin and adiponectin concentrations were determined by Luminex^® ^Multiplex technology (Luminex Corporation, Austin, TX) using Multiplex Immunoassays (Millipore, Amsterdam, The Netherlands) [23]. RNA was extracted from adipose tissue using Tri reagent (Sigma-Aldrich, St. Louis, MO) and subsequently converted into cDNA by a reverse transcription procedure using M-MLV and random primers according to the manufacturer's protocol (Sigma-Aldrich). For quantitative PCR (qPCR), cDNA was amplified using the appropriate primers and probes. The sequences for all primers and probes are given in Additional File 1 Table S2, and those that have been published are deposited at RTprimerdB http://www.rtprimerdb.org/. All mRNA levels were calculated relative to the expression of *cyclophilin *and normalized for expression levels of chow-fed *CB_1_^+/+ ^*mice.

### Indirect calorimetry

Homecage-housed animals were placed in an indirect calorimeter chamber with free access to food and drinking water. During 24 hours, gas exchange measurements were performed using an eight-channel open flow system. Flow rates were measured and controlled by a mass flow controller. O_2 _and CO_2 _concentrations of dried inlet and outlet air from each chamber were measured at 10-minute intervals using a paramagnetic O_2 _detector and an infrared CO_2 _detector. Estimated energy expenditure in kcal/24 hours was calculated using the following equation, which was derived from [24,25]: (24 * (16.18*VO_2_*0.001) + (5.02*VCO_2_*0.001))/4.184, with VO_2 _and VCO_2 _expressed in mL/h.

Fat and carbohydrate oxidation in mg/h were calculated using the following equation, which was derived from [26]:

Fat oxidation: 38.461*VO_2 _- VCO_2_, with VO_2 _and VCO_2 _expressed in mol/h.

Carbohydrate oxidation: 94.017*VCO_2 _- 66.239*VO_2_, with VO_2 _and VCO_2 _expressed in mol/h.

Data were calculated per animal or normalized for total lean body mass, which was determined by carcass analysis. Total lean mass represents the actual dry lean mass, and was calculated by adding the lean masses from intestine, organs, skin and carcass. Raw calorimetry- and detailed substrate utilization data are presented in Additional File 1 Figure S1A-S1C and Additional File 1 Table S3, respectively.

### Determination of de novo lipogenesis and chain elongation in adipose tissue

The assessment of lipogenic fluxes and chain elongation is derived from the enrichment of the adipose tissue acetyl-CoA precursor pool used for fatty acid synthesis using non-radioactive steady state isotope labelling. Lipogenic fluxes were calculated from the incorporation of ^13^C-labelled acetate into palmitate, stearate and oleate molecules within the adipose tissue [27,28]. In order to determine these parameters, animals received sodium [1-^13^C]-acetate (99 atom %, Isotec/Sigma-Aldrich) *via *the drinking water (2%) during the final 72 hours of the dietary period. After a postprandial fast, mice were sacrificed by cardiac puncture under isoflurane anaesthesia. Epididymal adipose tissue was quickly removed, snap-frozen in liquid nitrogen and stored at -80°C. Lipids were hydrolyzed in HCl/acetonitrile (1:22 v/v) for 45 minutes at 100°C. Fatty acids were extracted in hexane and derivatized for 15 minutes at room temperature using α-Br-2,3,4,5,6-pentafluorobenzyl (PFB)/acetonitrile/triethanolamine (1:6:2 v/v). Derivatization was stopped by adding HCl and fatty acid-PFB derivatives were extracted in hexane. The fatty acid-PFB mass isotopomer distributions were measured using an Agilent 5975 series GC/MSD (Agilent Technologies, Santa Clara, CA). Gas chromatography was performed using a ZB-1 column (Phenomenex, Torrance, CA). Mass spectrometry analysis was performed by electron capture negative ionization using methane as moderating gas.

The normalized mass isotopomer distributions measured by GC-MS (m_0_-m_x_) were corrected for natural abundance of ^13^C by multiple linear regression [29] to obtain the excess fractional distribution of mass isotopomers (M_0_-M_x_) due to incorporation of [1-^13^C]-acetate. This distribution was used in mass isotopomer distribution analysis (MIDA) algorithms to calculate the acetyl-CoA precursor pool enrichment (*p*_acetate_), fractional palmitate synthesis rates (f_C16:0_) and the fraction of stearate and oleate generated by elongation of *de novo *synthesized palmitate (f_C18:0/1(C16DNL)_), or by elongation of pre-existing palmitate (f_C18:0/1(C16PE)_) as described [28].

### Lipolytic activity

Following the postprandial fast, a baseline blood sample was drawn by retro-orbital bleeding under isoflurane anaesthesia into heparinized capillaries. Mice subsequently received an intra-orbital injection of heparin in saline (0.1 U/g body weight). After 10 minutes, a post-heparin blood sample was drawn by retro-orbital bleeding. Mice were sacrificed by cardiac puncture and blood was centrifuged (4000 × g for 10 minutes at 4°C). Total lipase activities were determined by incubating 10 μL of plasma with 200 μL of ultrasonified substrate containing 1 mL Triton X-100 (1%), 1 mL Tris.HCl (1 M), 2 mL of heat-inactivated human serum, 2 mL of fat-free BSA (10%), 42 mg triolein and 5 μL glycerol-tri-9,10(n)-[^3^H]- oleate (5 mCi/mL), with or without addition of 50 μL NaCl (5 M) to block LPL activity. After 30-min incubation at 37°C, lipolysis was stopped by adding 3.25 mL of heptane/methanol/chloroform (100:128:137, vol/vol/vol) and 1 mL of 0.1 M K_2_CO_3_. After centrifugation for 15 min at 3,600 rpm at room temperature, extracted hydrolyzed fatty acids were quantified by scintillation counting. Lipase activities were calculated according to the formula: [disintegrations per second (dps) sample - dps blank]/dps 200 μL LPL-substrate * factor, in which the factor = {2.45 (volume aqueous phase) * 2.85 (total added free fatty acids in micromoles)/[0.76 (extraction efficiency) * 0.5 (reaction time in hours) * 0.01 (plasma volume in mL)]}. Postheparin LPL activity was calculated by subtracting postheparin hepatic lipase activity (i.e., lipase activity inhibited by 1 M NaCl) from the total postheparin lipase activity.

Adipose tissue specific LPL activity was determined in biopsies collected after a postprandial fast. Tissue was first homogenized in 1 mL of a buffer containing sucrose (0.25 M), EDTA (1 mM), Tris.HCl (10 mM) and Deoxycholate (12 mM), pH 7.4 [30]. Tissue homogenates were centrifuged (20 min at 12000 × g for at 4°C) and the fraction between the upper fat layer and the bottom sediment was collected. Lipase activities were determined as described using 50 μl of tissue fraction. LPL activity was calculated by subtracting lipolytic activity determined in a final NaCl concentration of 0.83 M (non-LPL) from total lipolytic activity measured without NaCl. Data obtained in adipose tissue were normalized for protein concentration [30].

### Statistics

All data are presented as mean values ± SEM. Statistical analysis was performed using SPSS for Windows software (SPSS 16.0, Chicago, IL, USA). All parameters were first tested for equality of variances by Levene's test. Then, full-factorial General Linear Model analysis was performed to test for overall changes caused by diet or genotype, or for interactions between diet and genotype. Data were therefore initially analysed with a large statistical power to analyse overall effects, using groups of n > 10. For the overall dietary effects, Bonferoni post-hoc analysis was applied to correct for multiple comparisons. Outcome of general linear model analysis is given in the legends. Independent Student t-tests were run to analyse differences between genotypes within diets, and between dietary groups within genotypes. In the few cases where data transformation did not result in equality of variance, non-parametric analysis was performed. The null hypothesis was rejected at the 0.05 level of probability. Fat cell areas were considered significantly different between groups if the 95%-confidence intervals of the EC50 values obtained by PCRF analysis did not overlap.

## Results

### The resistance against high-fat diet-induced adiposity in CB_1_^-/- ^mice is associated with increased energy expenditure and reduced adipocyte size

**Figure 1:**
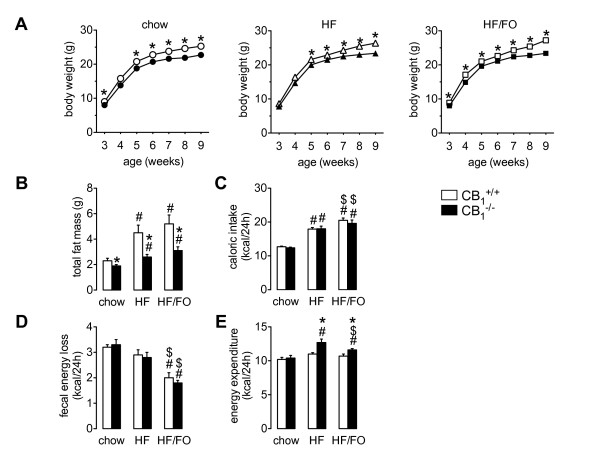
**Body weight, fat mass and energy balance in *CB_1_^+/+ ^*and *CB_1_^-/- ^*mice fed chow, a HF or a HF/FO diet during 6 weeks**. A, Body weight development during the dietary intervention. B, Total fat mass at the end of the dietary intervention. C, Caloric intake, determined by manual food weighing, and averaged over the third to fifth week of dietary intervention. D, Fecal energy loss and E, Energy expenditure, derived from indirect calorimetry. Open symbols/bars, *CB_1_^+/+ ^*mice; closed symbols/bars, *CB_1_^-/- ^*mice. Values are given as means ± SEM for *n *= 5-13; # *p *< 0.05 compared to chow group of the same genotype; $ *p *< 0.05 compared to HF group of the same genotype, * *p *< 0.05 *CB_1_^-/- ^vs*. *CB_1_^+/+ ^*(Student t-test). General linear model analysis revealed overall effects for the following parameters (*p *< 0.05): Genotype: body weight at every timepoint, total fat mass, 24 h-energy expenditure. Chow versus HF: total fat mass, caloric intake, 24 h-energy expenditure. Chow versus HF/FO: total fat mass, caloric intake, fecal energy loss. HF versus HF/FO: caloric intake, fecal energy loss.

We first evaluated the adiposity-inducing effects of two high-fat diets in young *CB_1_^+/+ ^*and *CB_1_^-/- ^*mice. Immediately after weaning, a 6-week dietary intervention was started during which animals were fed chow, HF or HF/FO diets. Body weight development during this intervention is presented in Figure 1A. We observed a steep increase in body weight during the first weeks of the dietary intervention in all groups, which likely indicates growth. Body weight development in this study therefore reflects both growth and increasing adiposity. Body mass was however always lower in *CB_1_*-deficient mice as compared to wild-type littermates, irrespective of the diet they received (Figure 1A). During the final weeks of the intervention, body weight curves of *CB_1_^+/+ ^*and *CB_1_^-/- ^*mice diverged, particularly in the HF and HF/FO group (Figure 1A). To assess to what extent the difference in body weight reflected changes in adiposity, we quantified the fat mass after finalisation of the dietary intervention. As expected, HF and HF/FO feeding resulted in a comparable increase in fat mass compared to chow in *CB_1_^+/+ ^*mice, while *CB_1_^-/- ^*mice remained relatively lean on the HF and HF/FO diets (Figure 1B). Whole-body carcass analysis revealed that, with the exception of brown fat, all adipose tissue depots contributed to the diet-induced adiposity (Table 1). Caloric intake was significantly higher in HF and HF/FO fed *CB_1_^+/+ ^*and *CB_1_^-/- ^*mice as compared to chow-fed controls (Figure 1C). The resistance to adiposity in *CB_1_^-/- ^*mice was however independent of caloric intake since we did not observe differences in energy consumption between *CB_1_^+/+ ^*and *CB_1_^-/- ^*mice during the dietary interventions (Figure 1C). Fecal energy loss was also similar in *CB_1_^+/+ ^*and *CB_1_^-/- ^*mice, indicating that intestinal energy absorption was comparable (Figure 1D). Fish oil replacement, however, increased nutrient absorption in HF-fed mice, since fecal energy loss was reduced in *CB_1_^+/+ ^*and *CB_1_^-/- ^*mice that were fed the HF/FO diet. A linear relationship between body weight and energy expenditure was observed for chow-fed *CB_1_^+/+ ^*and *CB_1_^-/- ^*mice only (data not shown), indicating that the increase in body weight upon HF and HF/FO feeding was not accompanied by elevated energy expenditure in this study. Nevertheless, we found that estimated energy expenditure was clearly and significantly increased in HF and HF/FO-fed *CB_1_*-deficient mice as compared to wild-type controls (Figure 1E and Additional File 1 Table S3). Altogether, analysis of the different components of energy balance (Figures 1C-E) reveals equal energy intake and -expenditure in chow-fed *CB_1_^+/+ ^*and *CB_1_^-/- ^*mice. On the other hand, HF and HF/FO feeding induced a positive energy balance, which is less marked in *CB_1_^-/- ^*versus *CB_1_^+/+ ^*mice, as a result of increased energy expenditure. Interestingly, chow-fed *CB_1_^-/- ^*mice exhibited higher fat oxidation rates in the dark phase (Additional File 1 Table S3), which likely explains the lower fat mass as compared to *CB_1_^+/+ ^*mice (Figure 1B and Table 1).

**Table 1:** Body composition and plasma metabolite concentrations in *CB_1_^+/+ ^*and *CB_1_^-/- ^*mice fed chow, a HF or a HF/FO diet during 6 weeks

	chow		HF		HF/FO	
	*CB_1_^+/+^*	*CB_1_^-/-^*	*CB_1_^+/+^*	*CB_1_^-/-^*	*CB_1_^+/+^*	*CB_1_^-/-^*
						
Subcutaneous fat (g)	0.9 ± 0.1	0.7 ± 0.0	2.1 ± 0.3#	1.1 ± 0.1#*	2.5 ± 0.4#	1.4 ± 0.1#*
Epididymal fat (mg)	319 ± 40	227 ± 19	716 ± 113#	361 ± 34#*	644 ± 85#	406 ± 64#*
Retroperitoneal fat (mg)	116 ± 17	66 ± 6*	217 ± 33#	108 ± 7#*	300 ± 54#	145 ± 19#*
Brown fat (mg)	45 ± 6	41 ± 5	47 ± 6	36 ± 3	52 ± 5	36 ± 5
Mesenteric fat (mg)	194 ± 18	148 ± 12	303 ± 36#	181 ± 11*	336 ± 47#	190 ± 14*
Muscular fat (g)	0.6 ± 0.0	0.5 ± 0.0*	1.0 ± 0.1#	0.7 ± 0.0#*	1.1 ± 0.1#	0.8 ± 0.0#*
Organ fat (mg)	113 ± 15	103 ± 14	176 ± 28	161 ± 15#	238 ± 30#	133 ± 14*
Lean mass (g)	5.2 ± 0.6	4.6 ± 0.1*	5.0 ± 0.1	4.7 ± 0.1	5.0 ± 0.1	4.7 ± 0.1

**Plasma metabolites and adipokines**						
Triglycerides (μM)	333 ± 31	245 ± 31	277 ± 29	275 ± 33	240 ± 63	183 ± 19
Cholesterol (mM)	1.3 ± 0.2	0.9 ± 0.1	1.9 ± 0.1#	1.8 ± 0.1#	1.4 ± 0.1$	1.3 ± 0.1#$
Non-esterified fatty acids (μM)	607 ± 32	391 ± 34*	586 ± 40	588 ± 51#	464 ± 38#	372 ± 26$
Blood glucose (mM)	9.3 ± 0.5	7.7 ± 0.8	9.1 ± 0.3	7.4 ± 0.4*	9.0 ± 0.4	8.1 ± 0.3
Insulin (pg/mL)	334 ± 53	356 ± 86	325 ± 39	270 ± 64	371 ± 82	300 ± 84
Leptin (ng/mL)	0.7 ± 0.1	0.6 ± 0.1$	2.1 ± 0.5#*	1.0 ± 0.2#*	2.2 ± 0.5#	1.7 ± 0.3#
Adiponectin (μg/mL)	14.3 ± 1.3	18.5 ± 2.4	15.4 ± 1.5	17.0 ± 2.9	18.1 ± 2.2	19.1 ± 2.2
Resistin (ng/mL)	2.3 ± 0.2	2.5 ± 0.4	3.0 ± 0.5	2.9 ± 0.3	2.1 ± 0.2	2.6 ± 0.4
						

Values are given as means ± SEM for *n *= 6-8; # *p *< 0.05 compared to chow group of the same genotype; $ *p *< 0.05 compared to HF group of the same genotype, * *p *< 0.05 *CB_1_^-/- ^vs*. *CB_1_^+/+ ^*(Student t-test).

General linear model analysis revealed overall effects for the following parameters (*p *< 0.05):

Genotype: subcutaneous/epidydimal/retroperitoneal/brown/mesenteric/muscular/organ fat mass, lean mass, non-esterified fatty acid levels, glucose levels.

Chow versus HF: subcutaneous/epididymal/retroperitoneal/mesenteric/muscular/organ fat mass, leptin levels.

Chow versus HF/FO: subcutaneous/epididymal/retroperitoneal/mesenteric/muscular/organ fat mass, cholesterol levels, leptin levels.

HF versus HF/FO: non-esterified fatty acid levels.

Interaction between genotype and diet: non-esterified fatty acid levels.

**Figure 2:**
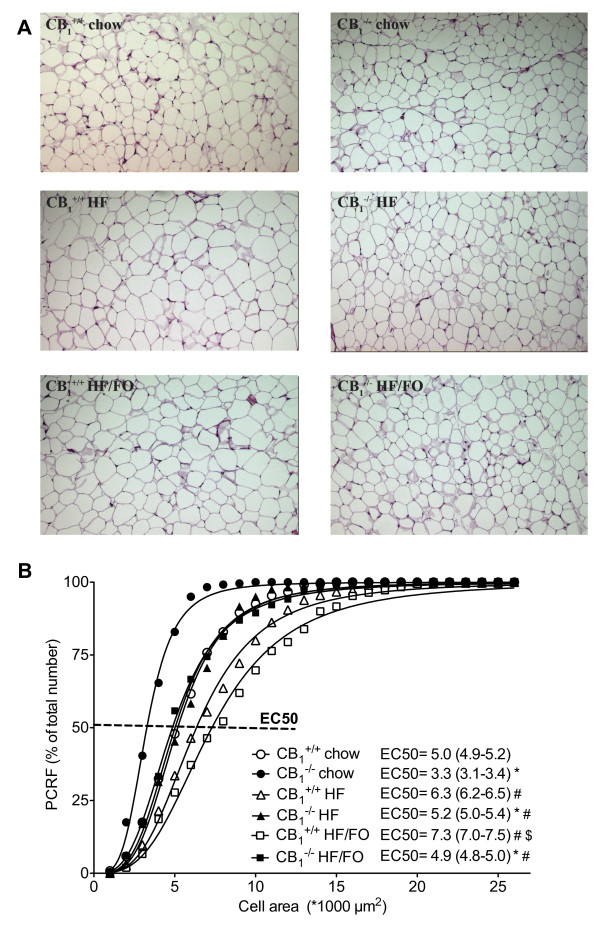
**Fat cell area in *CB_1_^+/+ ^*and *CB_1_^-/- ^*mice fed chow, a HF or a HF/FO diet during 6 weeks**. A, Representative pictures of 3 μm paraffin hematoxylin and eosin-stained sections (1 cm = 100 μm) and B, percent relative cumulative frequency (PCRF) curves of 240-420 fat cell areas from adipose tissue sections. Inset: EC50 values of the PCRF curves and their 95%-confidence intervals. Open symbols, *CB_1_^+/+ ^*mice; closed symbols, *CB_1_^-/- ^*mice. # *p *< 0.05 compared to chow group of the same genotype; $ *p *< 0.05 compared to HF group of the same genotype, * *p *< 0.05 *CB_1_^-/- ^vs*. *CB_1_^+/+ ^*(*p *< 0.05 in case of no overlap between EC50 95%-confidence intervals).

HF and HF/FO feeding led to an increase in adipocyte size in both *CB_1_^+/+ ^*and *CB_1_^-/- ^*mice (Figure 2A and 2B). Similar to body and fat mass, *CB_1_^-/- ^*mice always had smaller adipocytes (Figure 2A), and we observed a significant difference between the 95%-confidence intervals of the fat cell area EC50s in *CB_1_^-/- ^*versus *CB_1_^+/+ ^*mice fed either of the three diets (Figure 2B). Adipose tissue expression of *Faah*, *Pparγ2*, *C/ebpα*, *Ap2 *and *Adiponectin *were not affected by *CB_1 _*deficiency or HF and HF/FO feeding, while *Napepld *expression was increased in HF-fed mice of both genotypes (Table 2). Plasma triglyceride, insulin, adiponectin and resistin levels were comparable in all mice (Table 1). Cholesterol concentrations were increased in HF-fed *CB_1_^+/+ ^*and *CB_1_^-/- ^*mice as compared to those receiving chow, and normalized in HF/FO-fed mice (Table 1). Non-esterified fatty acid levels were significantly lower in chow- and HF/FO-fed *CB_1_-*deficient mice (Table 1). Glucose levels tended to be lower in *CB_1_^-/- ^*versus *CB_1_^+/+ ^*mice, however the difference reached statistical significance for HF-fed animals only (Table 1). Plasma leptin concentrations (Table 1) correlated to the degree of HF and HF/FO-induced adiposity (Figure 1B) in *CB_1_^+/+ ^*and *CB_1_^-/- ^*mice.

**Table 2:** Adipose tissue gene expression levels in *CB_1_^+/+ ^*and *CB_1_^-/- ^*mice fed chow, a HF or a HF/FO diet during 6 weeks

	chow		HF		HF/FO	
	*CB_1_^+/+^*	*CB_1_^-/-^*	*CB_1_^+/+^*	*CB_1_^-/-^*	*CB_1_^+/+^*	*CB_1_^-/-^*
						
*Endocannabinoid system*						
CB_1 _(Cnr1)	1.0 ± 0.1	ND	1.0 ± 0.1	ND	1.1 ± 0.2	ND
Napepld	1.0 ± 0.1	0.9 ± 0.1	1.4 ± 0.1#	1.3 ± 0.2#	1.2 ± 0.1	1.1 ± 0.1
Faah	1.0 ± 0.3	1.8 ± 0.5	1.1 ± 0.6	1.1 ± 0.4	1.0 ± 0.5	1.3 ± 0.6
*Adipocyte proliferation and differentiation*						
Pparγ2 (Pparg)	1.0 ± 0.2	1.0 ± 0.1	1.4 ± 0.1	1.2 ± 0.3	1.0 ± 0.1$	0.9 ± 0.1
C/ebpα (Cebpa)	1.0 ± 0.1	1.0 ± 0.1	1.2 ± 0.0	1.3 ± 0.3	1.0 ± 0.1	0.9 ± 0.1
Ap2 (Fabp4)	1.0 ± 0.1	0.9 ± 0.1	1.5 ± 0.0#	1.2 ± 0.2	1.4 ± 0.1	1.2 ± 0.1
Adiponectin (Adipoq)	1.0 ± 0.2	0.9 ± 0.2	1.3 ± 0.1	1.3 ± 0.4	1.3 ± 0.1	1.0 ± 0.1
*Trigyceride synthesis*						
Srebp-1c (Srebf1)	1.0 ± 0.1	1.2 ± 0.1	1.5 ± 0.1#	1.8 ± 0.4	1.1 ± 0.0$	1.1 ± 0.0
Acc1 (Acaca)	1.0 ± 0.2	1.1 ± 0.3	0.9 ± 0.1	0.9 ± 0.3	0.6 ± 0.1$	0.5 ± 0.1#
Fas (Fasn)	1.0 ± 0.2	1.1 ± 0.2	1.1 ± 0.1	1.0 ± 0.3	0.7 ± 0.1$	0.4 ± 0.0#
Scd1	1.0 ± 0.1	0.9 ± 0.1	2.8 ± 0.1#	3.1 ± 0.7#	0.9 ± 0.1$	1.1 ± 0.1$
Pepck (Pck1)	1.0 ± 0.2	0.9 ± 0.1	1.0 ± 0.1	1.3 ± 0.4	1.1 ± 0.1	0.9 ± 0.1
*Fatty acid uptake and transport *						
Cd36	1.0 ± 0.1	1.1 ± 0.1	1.1 ± 0.0	1.2 ± 0.1	1.4 ± 0.2	1.1 ± 0.1
Fatp4 (Slc27a4)	1.0 ± 0.1	1.0 ± 0.1	1.2 ± 0.0	1.4 ± 0.2	1.2 ± 0.1	1.1 ± 0.1
*Fatty acid hydrolysis *						
Lpl	1.0 ± 0.3	0.9 ± 0.2	1.2 ± 0.1	0.9 ± 0.2	1.4 ± 0.2	1.0 ± 0.1
Gpihbp1	1.0 ± 0.1	1.2 ± 0.1	1.2 ± 0.1	1.2 ± 0.1	1.3 ± 0.1	0.9 ± 0.0
Angptl3	1.0 ± 0.1	0.9 ± 0.1	1.2 ± 0.1	1.1 ± 0.1	1.0 ± 0.1	1.0 ± 0.0
Angptl4	1.0 ± 0.1	1.0 ± 0.1	1.3 ± 0.1	1.2 ± 0.1	1.5 ± 0.1	1.1 ± 0.1
Apoc1	1.0 ± 0.2	1.2 ± 0.1	1.1 ± 0.0	1.1 ± 0.0	0.7 ± 0.1$	0.9 ± 0.1
Apoc3	1.0 ± 0.2	0.7 ± 0.1	0.6 ± 0.1	0.4 ± 0.0	0.2 ± 0.0$	0.2 ± 0.0#
*Macrophage infiltration*						
Cd68	1.0 ± 0.1	1.0 ± 0.1	1.2 ± 0.0	1.0 ± 0.1	1.3 ± 0.1	1.0 ± 0.1
*Adipocyte lipolysis*						
Hsl (Lipe)	1.0 ± 0.2	1.1 ± 0.2	1.2 ± 0.1	1.0 ± 0.2	1.2 ± 0.1	1.1 ± 0.1
Atgl (Pnpla2)	1.0 ± 0.2	1.1 ± 0.2	1.5 ± 0.2	1.4 ± 0.4	1.2 ± 0.1	1.2 ± 0.2
*Fatty acid oxidation*						
Cpt1a	1.0 ± 0.1	1.2 ± 0.2	1.6 ± 0.2#	1.2 ± 0.2	1.3 ± 0.1#	1.1 ± 0.1

Expression levels were normalized to cyclophilin expression and values are given as means ± SEM for *n *= 4-8; # *p *< 0.05 compared to chow group of the same genotype, $ *p *< 0.05 compared to HF group of the same genotype (Student t-test). ND, non-detectable.

General linear model analysis revealed overall effects for the following parameters (*p *< 0.05):

Chow versus HF: aP2, Scd1, Srebp-1c.

Chow versus HF/FO: Fas.

HF versus HF/FO: Fas, Scd1, Srebp-1c.

### Fatty acid synthesis in adipose tissue is not affected in CB_1_^-/- ^mice

**Figure 3:**
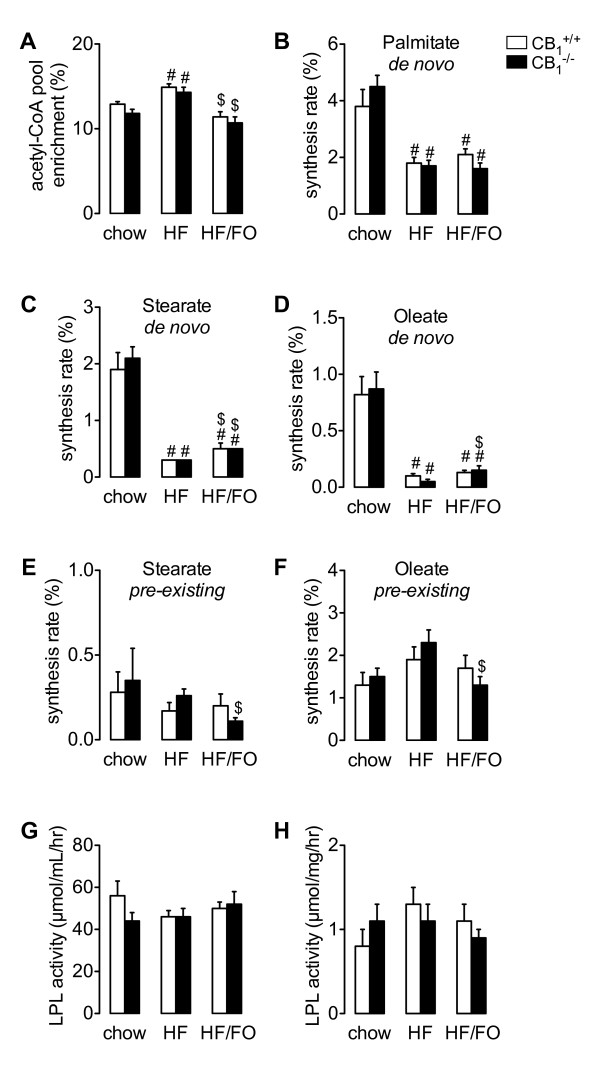
**Fractional fatty acid synthesis rates and lipolytic activities in *CB_1_^+/+ ^*and *CB_1_^-/- ^*mice fed chow, a HF or a HF/FO diet during 6 weeks**. A, Acetyl-CoA precursor pool enrichment. B, *De novo *palmitate synthesis. C *De novo *stearate synthesis. D, *De novo *oleate synthesis. E, Stearate synthesis from pre-existing palmitate. F, Oleate synthesis from pre-existing palmitate. G, Post-heparin plasma LPL activity and H, Adipose tissue-derived LPL activity normalized for protein content. Open bars, *CB_1_^+/+ ^*mice; closed bars, *CB_1_^-/- ^*mice. Values are given as means ± SEM for *n *= 4-7; # *p *< 0.05 compared to chow group of the same genotype; $ *p *< 0.05 compared to HF group of the same genotype (Student t-test). General linear model analysis revealed overall effects for the following parameters (*p *< 0.05): Chow versus HF: acetyl-CoA pool enrichment, *de novo *palmitate/stearate/oleate synthesis. Chow versus HF/FO: *de novo *palmitate/stearate synthesis. HF versus HF/FO: acetyl-CoA pool enrichment, *de novo *stearate/oleate synthesis.

To assess whether lipogenesis was reduced in *CB_1_^-/- ^*mice, we determined fatty acid synthesis in adipose tissue using ^13^C-acetate incorporation. Acetyl-CoA pool enrichments were increased in HF-fed *CB_1_^+/+ ^*and *CB_1_^-/- ^*mice as compared to chow-fed animals (Figure 3A), and normalized by FO replacement in both genotypes. No differences between *CB_1_^+/+ ^*and *CB_1_^-/- ^*mice were observed. *De novo *palmitate synthesis was similar in *CB_1_^+/+ ^*and *CB_1_^-/- ^*mice, but was reduced in mice fed the HF and HF/FO diets as compared to chow-fed animals (Figure 3B). Similarly, fractional synthesis of stearate and oleate via *de novo lipogenesis *was reduced in *CB_1_^+/+ ^*and *CB_1_^-/- ^*mice fed the HF and HF/FO diets as compared to chow-fed animals (Figure 3C and 3D) with no differences between *CB_1_^+/+ ^*and *CB_1_^-/- ^*mice. Compared to HF-fed mice, *de novo *stearate synthesis was slightly increased in HF/FO-fed mice of both genotypes (Figure 3C). The unaltered fatty acid synthesis rates in *CB_1_^-/- ^*mice were supported by gene expression analysis in adipose tissue: we did not observe any changes in lipogenic gene expression between *CB_1_^+/+ ^*and *CB_1_^-/- ^*mice under the different dietary conditions (Table 2). HF feeding did not affect *Acc1, Fas *and *Pepck *(Table 2) expression. HF feeding induced *Scd1 *expression in both *CB_1_^+/+ ^*and *CB_1_^-/- ^*mice while we observed a tendency for an increased *Srebp-1c *expression. These inductions were normalized in HF/FO-fed *CB_1_^+/+ ^*and *CB_1_^-/- ^*mice (Table 2). HF/FO feeding also reduced *Acc *and *Fas *expression levels as compared to HF (Table 2). Fatty acids are toxic molecules, which are immediately processed after they enter cells to prevent apoptosis [31]. Chain-elongation and desaturation of pre-existing palmitate may therefore be used as a measure for fatty acid entry [31]. In contrast to the reduced lipogenic fluxes from elongation of *de novo *synthesized palmitate (Figure 3B-3D), we did not observe any consistent differences in synthesis of stearate (Figure 3E) and oleate (Figure 3F) from elongation of pre-existing palmitate between *CB_1_^+/+ ^*and *CB_1_^-/- ^*mice. Reduced fatty acid influx therefore does not explain the protection against HF and HF/FO-induced adiposity in *CB_1_*-deficient mice. This is further supported to the unchanged mRNA levels of the fatty acid transporters *Cd36 *and *Fatp4 *in these animals (Table 2).

### Lipolytic activity is maintained in CB_1_^-/- ^mice

CB_1_-receptor agonist treatment enhances lipolytic activity *in vitro *[5]. As a consequence, *CB_1 _*deficiency may result in a reduction of lipoprotein triglyceride lipolysis, thereby limiting fat storage in adipose tissue and as such protecting against adiposity. Yet, the expression of *Lpl *and its stimulatory factor *Gpihbp1 *[32] in adipose tissue were not affected by *CB_1 _*deficiency or HF and HF/FO feeding (Table 2). In parallel, we determined LPL activities in *CB_1_^+/+ ^*and *CB_1_^-/- ^*mice fed either of the three diets. In accordance with unaltered *Lpl *and *Gpihbp1 *mRNA levels, we observed that both post-heparin plasma LPL activity and adipose tissue-derived LPL-activity were similar in all groups (Figure 3G and 3H). The expression levels of the LPL inhibitory proteins Angptl3, Angplt4, ApoC1 and ApoC3 [32,33] were also unchanged between genotypes (Table 2). Because macrophage products have been shown to inhibit adipocyte differentiation [34], we determined CD68 macrophage antigen expression. Consistent with the unaltered mRNA levels of adipocyte differentiation/proliferation markers, we found no significant differences in *Cd68 *expression between *CB_1_*-deficient and wild-type mice (Table 2). Finally, we did not observe significant differences in adipose tissue expression of *Hsl*, *Atgl *and *Cpt1a *between *CB_1_^+/+ ^*and *CB_1_^-/- ^*mice (Table 2), suggesting that fatty acid release and -oxidation were also not affected by *CB_1 _*deficiency.

## Discussion

Overactivity and/or dysregulation of the EC system potentially contribute to the development of obesity [7,11,14]. Several *in vitro *studies point to the involvement of the CB_1_-receptor in controlling adipocyte development and metabolism [5-13]. Here, we report that the protection against high-fat diet induced adiposity and a concomitant reduced fat cell size in *CB_1_*-deficient mice are not related to alterations in adipocyte differentiation and proliferation markers, or to changes lipogenic fluxes and lipoprotein lipolysis in fat tissue.

Diet-induced adiposity was prevented in *CB_1_^-/- ^*mice as compared to wild-type littermates. Analysis of the energy balance in chow, HF and HF/FO-fed animals revealed that *CB_1 _*deficiency did not impact on energy intake. Consistent with previous findings [35], no major effects on food intake were observed. Although both HF and HF/FO feeding increased intestinal fatty acid uptake, this occurred to a similar extent in *CB_1_^-/- ^*and *CB_1_^+/+ ^*mice. Interestingly, isocaloric replacement of the saturated fat in the HF diet for PUFA provoked an additional increase in intestinal fatty acid uptake. This most likely resulted from an upregulation of intestinal fatty acid transport proteins and a subsequent increase in lipid uptake [36]. On the other hand, we observed that energy expenditure was increased in HF and HF/FO-fed *CB_1_^-/- ^*mice as compared to wild-type littermates. Reduced diet-induced adiposity upon *CB_1 _*ablation was therefore associated with a less positive energy balance in the current study.

Impaired *CB_1_*-signalling has been shown to protect against high-fat diet-induced obesity in adult mice [16,35], however, the adipocyte-specific consequences in this context had not been evaluated. The ECS has been implicated in the regulation of adipocyte proliferation and differentiation *in vitro *[5-13] and *CB_1_*-deficient mice are retarded in growth from an early age on [5]. We therefore decided to study the metabolic consequences of high-fat feeding in young *CB_1_^-/- ^*mice. Six weeks of HF and HF/FO feeding induced adiposity in *CB_1_^+/+ ^*mice, which was characterized by an increased fat mass and adipocyte size. This effect was clearly blunted in *CB_1_^-/- ^*mice, although these mice did become more obese on the HF and HF/FO diets as compared to chow-fed *CB_1_^-/- ^*mice. Gene expression analysis indicated that this was not due to a lower expression of adipocyte differentiation and proliferation markers in *CB_1_^-/- ^*mice, since we did not observe differences in *Pparγ2*, *Cebp*/*α *and *Ap2 *mRNA levels. The expression of these genes was also not different prior to the start of the dietary interventions (Additional File 1 Figure S1D).

PPARγ and C/EBPα are two master transcription factors which play key roles in controlling adipocytes differentiation and proliferation, as well as adiponectin gene transcription [37,38]. We did not observe differences in *adiponectin *mRNA levels, indicating that, in parallel to the unaltered expression levels of PPARγ and C/EBPα, the transcriptional activity of these regulators was not impaired by *CB_1 _*deficiency. In accordance with these findings, we observed that plasma adiponectin concentrations remained unchanged among the different groups studied. Because of the relatively short timeframe of the dietary intervention, HF and HF/FO feeding did not yet alter adiponectin levels as compared to chow-fed mice [39]. Although CB_1_-receptor activity has been proposed to modulate adiponectin expression and release [7,11], pharmacological CB_1_-receptor activation and blockade do not directly affect adiponectin expression in cultured adipocytes [12,40]. The reported effects on adiponectin expression and -secretion are therefore rather related to acute changes in food intake [41]. Consistent with the unchanged adiponectin levels and similar to what has been found by others [35], voluntary caloric intake was not different between *CB_1_^+/+ ^*and *CB_1_*^-/- ^mice.

Pharmacological CB_1_-receptor activation promotes lipid storage in adipocytes [6,11-13], in parallel to elevated expression levels of lipogenic genes in fat tissue [6]. We therefore quantified the lipogenic flux *in vivo *in adipose tissue of *CB_1_^+/+ ^*and *CB_1_^-/- ^*mice. Fatty acid synthesis rates were not affected by *CB_1 _*deficiency, in line with unaltered expression levels of lipogenic genes. In both genotypes, HF and HF/FO feeding reduced *de novo *lipogenesis (indicated by lower fractional palmitate synthesis as well as by reduced stearate and oleate synthesis from *de novo *synthesized palmitate). This is in agreement with a report that shows reduced fatty acid synthesis from glucose in the fat tissue of high-fat fed mice compared to animals receiving a low-fat diet [42]. Thus, high-fat diet-induced adiposity is caused by increased storage and elongation of dietary fatty acids. The latter process was however not affected by *CB_1 _*deficiency in the current study, since we found that fatty acid elongation of pre-existing palmitate was maintained in *CB_1_^-/- ^*mice. The obesity-resistant phenotype of these animals can therefore not be ascribed to a defect in fatty acid synthesis or -elongation.

Lipid supply to organs and tissues requires the activity of lipases. Adipocyte-specific LPL activity directs fatty acids to the adipose tissue, thereby enabling their storage. To assess whether the protection against high-fat diet-induced adiposity in *CB_1_^-/- ^*mice was secondary to a reduced lipolytic activity, we determined whole-body and adipose tissue-specific LPL activity. *CB_1_^+/+ ^*and *CB_1_*^-/- ^mice fed either of the three diets exhibited comparable LPL activities. Elevated plasma EC concentrations have been associated with increased LPL activity [43]. *In vitro *studies have furthermore shown that CB_1_-receptor antagonism inhibits LPL activity in adipocytes, however, only when these cells are co-treated with a CB_1_-receptor agonist [5]. Together, these findings indicate that CB_1_-receptor blockade only impacts on LPL activity under conditions of active ECS signalling. Consistent with previous work [35], we did not observe an increase in LPL activity upon high-fat feeding. LPL activity was furthermore not different between *CB_1_^+/+ ^*and *CB_1_*^-/- ^mice, suggesting that the HF diet did not increase CB_1_-mediated LPL activity, which rendered this process insensitive to *CB_1 _*ablation. In further support of this were the unchanged expression levels of LPL and its regulators among the different groups. Thus, the reduced fat stores in *CB_1_*-deficient mice were not related to impaired LPL-mediated lipolysis. The unaltered expression levels of *Hsl *and *Atgl *and the lack of an increase in plasma non-esterified fatty acid levels in *CB_1_*-deficient mice furthermore argues against increased fatty acid release from adipose tissue in these animals.

Although several studies have implicated the ECS in control of adipocyte differentiation/proliferation, lipogenesis and lipolysis [5-13], the current work excludes the possibility that these processes contribute to the development of obesity through CB_1_. One of the explanations for these discrepancies presumably lies in the acute and transient nature of responses to pharmacological treatments [44], whereas our knockout mice rather provide a model of sustained impaired CB_1 _activity. The adaptations of adipocyte biology in response to altered EC composition, on the other hand, are most likely mediated by CB_1_-independent mechanisms. Overall, our data indicate that the reported physiological changes in adipocyte biology that occur in response to altered ECS activity cannot explain the phenotype of *CB_1_^-/- ^*mice in the long term.

Reduced diet-induced fat storage in *CB_1_^-/- ^*mice was primarily related to a less positive energy balance as compared to their wild type littermates in this study. Recent reports show that decreased ECS signalling promotes energy dissipation as heat. CB_1_-receptor blockade induces a mitochondria-rich, thermogenic phenotype in adipocytes, characterized by increased mitochondrial uncoupling [45-48]. CB_1_-agonists, on the other hand, impair mitochondrial biogenesis and -uncoupling [6,49]. Therefore, both oxidative and non-oxidative energy expenditure presumably contribute to the protection against diet-induced adiposity *CB_1_*-deficient mice.

## Conclusions

This study shows that the obesity-resistant phenotype of *CB_1_*-deficient mice is not due to changes in adipose tissue differentiation and -proliferation, lipogenesis or LPL-activity *in vivo*. Wild-type and *CB_1_*-deficient mice exhibited similar energy intakes. Energy expenditure, on the other hand, was increased in *CB_1_^-/- ^*mice upon high-fat feeding, resulting in lower adiposity and decreased fat cell size. We therefore propose that the protection against diet-induced adiposity in *CB_1_^-/- ^*mice is not related to functional alterations in fat metabolism *per se*, but rather results from increased energy loss by oxidative and potentially non-oxidative pathways.

## List of abbreviations

Acc1: acetyl-CoA carboxylase 1; AEA: anandamide; 2-AG, 2-arachidonoyl glycerol; Angptl: Angiopoietin-like protein; Ap2: fatty acid-binding protein 4; ApoC: apolipoprotein C; Atgl: adipose triglyceride lipase; CB_1: _cannabinoid receptor 1 (Cnr1); Cd36: fatty acid transporter; Cd68: macrophage antigen 68; C/ebpα: CCAAT enhancer binding protein α; Cpt1a: carnitine palmitoyl transferase 1a; EC: endocannabinoid; ECS: endocannabinoid system; Faah: fatty acid amide hydrolase; Fas: fatty acid synthase; Fatp4: fatty acid transport protein 4; Gpihbp1: glycosylphosphatidylinositol anchored high density lipoprotein binding protein 1; HF: high-fat; HF/FO: high-fat/fish oil; Hsl: hormone sensitive lipase; Lpl: lipoprotein lipase; MIDA: mass isotopomer distribution analysis; Napepld: N-acyl phosphatidylethanolamine phospholipase D; PCRF: percent relative cumulative frequency; Pepck: phosphoenolpyruvate carboxykinase; PFB: pentafluorobenzyl; Pparγ2: peroxisome proliferator-activated receptor gamma isoform 2; PUFA: polyunsaturated fatty acid; qPCR: quantitative PCR; Scd1: stearoyl-CoA desaturase 1; Srebp-1c: sterol regulatory element binding protein 1c.

## Competing interests

The authors declare that they have no competing interests.

## Authors' contributions

MHO and AHK were involved in the acquisition and analysis of the data, participated in the design of the study and drafted the manuscript. PTB, TB, AB and THD were responsible for data acquisition. VWB and PJJS and contributed to interpretation of the data and critical revision of the manuscript. FK and GJD conceived of the study, participated in its design and coordination, and critically revised the manuscript. All authors read and approved the final manuscript.

## Supplementary Material

Additional file 1 **Table S1 Composition of experimental diets**. Table S2 Primer and probe sequences used for qPCR. Sequence and accessions numbers of qPCR primers and probes used in these study. Figure S1 (**A**) VO_2_, (**B**) VCO_2 _and (**C**) RER values during light and dark phases. Open symbols, *CB_1_^+/+ ^*mice; closed symbols, *CB_1_^-/- ^*mice. Values are given as means ± SEM for *n *= 5-7. (**D**) Gene expression levels in epididymal fat tissue of 3-week old *CB_1_^-/- ^*and *CB_1_^+/+ ^*mice receiving regular chow. Open bars, *CB_1_^+/+ ^*mice; closed bars, *CB_1_^-/- ^*mice. Values are given as means ± SEM for *n *= 4-8. Table S3 Detailed indirect calorimetry data in *CB_1_^+/+ ^*and *CB_1_^-/- ^*mice fed chow, a HF or a HF/FO diet during 6 weeks. Energy expenditure and substrate utilization during dark and light phases, expressed per mouse (upper part) or normalized for lean body mass (lower part). Click here for file
