# Supplementary material for: Resistance to diet-induced adiposity in cannabinoid receptor-1 deficient mice is not due to impaired adipocyte function
Source: Nutr Metab (Lond). 2011 Dec 27;8:93. doi: 10.1186/1743-7075-8-93 (PMC3307495; doi:10.1186/1743-7075-8-93)
Supplement: Additional file 1 — Table S1 Composition of experimental diets. Table S2 Primer and probe sequences used for qPCR. Sequence and accessions numbers of qPCR primers and probes used in these study. Figure S1 (A) VO2, (B) VCO2 and (C) RER values during light and dark phases. Open symbols, CB1+/+ mice; closed symbols, CB1-/- mice. Values are given as means ± SEM for n = 5-7. (D) Gene expression levels in epididymal fat tissue of 3-week old CB1-/- and CB1+/+ mice receiving regular chow. Open bars, CB1+/+ mice; closed bars, CB1-/- mice. Values are given as means ± SEM for n = 4-8. Table S3 Detailed indirect calorimetry data in CB1+/+ and CB1-/- mice fed chow, a HF or a HF/FO diet during 6 weeks. Energy expenditure and substrate utilization during dark and light phases, expressed per mouse (upper part) or normalized for lean body mass (lower part). [file 1743-7075-8-93-S1.PDF]

Additional File 1, Table S1. Composition experimental diets.

	**CHOW**	**HF**	**HF/FO**
Starch	363	147	147
Protein	211	201	201
Glucose	47	158	158
Fatty acids			
C14:0	0.5	12.2	16.1
C16:0	8.4	92.5	79.5
C16:1	0.7	11.5	18.0
C18:0	3.7	76.3	50.5
C18:1	13.7	133.2	101.0
C18:2	16.9	11.5	9.7
C18:3	1.9	2.9	15.2
C20-22	0.4	4.0	53.3

Values are given in g/kg.

Additional File 1, Table S2. Primer and probe sequences used for qPCR.

Gene	Sense	Antisense	Probe	Accession number
Adiponectin (Adipoq)	AGG ACA TCC TGG CCA CAA TG	CTT AGG ACC AAG AAG ACC TGC AT	CTC TCC AGG AGT GCC ATC TCT GCC A	NM_009605
Acc1 (Acaca)	CCA TCC AAA CAG AGG GAA CAT C	CTA CAT GAG TCA TGC CAT AGT GGT T	ACG CTA AAC AGA ATG TCC TTT GCC TCC AAC	NM_133360
Angptl3	CCC AGA GCA CAC AGA CCT	CAC CAC CAG CCA CCT GAG	AGC TGT CCC TTT GCT CTG TGA TTC CAT	NM_013913
Angptl4	AGA TCC AGC AAT TGT TCC AGA AG	AAG AGG TCT ATC TGG CTC TGA AGA TT	CCC AGC AGC AGA GAT ACC TAT CAA AGC AG	NM_020581
Ap2 (Fabp4)	CAC CAT CCG GTC AGA GAG TAC TT	TCT AGG GTT ATG ATG CTC TTC ACC T	CAT CGA ATT CCA CGC CCA GTT TGA	NM_024406
Apoc1	GGG CAG CCA TTG AAC ATA TCA	TTG CCA AAT GCC TCT GAG AAC	CCC GGG TCT TGG TCA AAA TTT CCT TC	NM_007469
Apoc3	CCA AGA CGG TCC AGG ATG C	ACT TGC TCC AGT AGC CTT TCA GG	CCA TCC AGC CCC TGG CCA CC	NM_023114
Atgl (Pnpla2)	AGC ATC TGC CAG TAT CTG GTG AT	CAC CTG CTC AGA CAG TCT GGA A	ATG GTC ACC CAA TTT CCT CTT GGC CC	NM_025802
Cb1 (Cnr1)	ACA AGC TTA TCA AGA CGG TGT TTG	TGC TCC TCA GAG CAT AGA TGA TG	CTC TGC CTG CTG AAC TCC ACC GTG	NM_007726
Cd36 (Fat)	GAT CGG AAC TGT GGG CTC AT	GGT TCC TTC TTC AAG GAC AAC TTC	AGA ATG CCT CCA AAC ACA GCC AGG AC	BC010262
Cd68	CAC TTC GGG CCA TGT TTC TC	AGG ACC AGG CCA ATG ATG AG	CAA CCG TGA CCA GTC CCT CTT GCT G	NM_009853

C/ebpα (Cebpa)	CCA AGA AGT CGG TGG ACA AGA A	AGG CGG TCA TTG TCA CTG GT	CGC AAC AAC ATC GCG GTG CG	NM_007678
Cpt1a	CTC AGT GGG AGC GAC TCT TCA	GGC CTC TGT GGT ACA CGA CAA	CCT GGG GAG GAG ACA GAC ACC ATC CAA C	NM_013495
Faah	CAG AAG CTG TGC TCT TTA CCT ACC	CAG ATA GGA GGT CAC ACA GTT GGT	CTT TGT TCA CTT CCC AGG CCT TTC CC	NM_010173
Fas (Fasn)	GGC ATC ATT GGG CAC TCC TT	GCT GCA AGC ACA GCC TCT CT	CCA TCT GCA TAG CCA CAG GCA ACC TC	NM_007988
Fatp4 (Slc27a4)	CCA GAC AAG GGT TTT ACA GAT AAG CT	ACC TGC TGT GCA CCA CAA TG	CGG GCA CCA CGG GGC TAC CC	NM_011989
Gpihbp1	GCG GAA CCG ACA AAG GTT AC	TGC CTC CCA CTG TCT TGA TG	CCA TGT GGT GTA CTG ATA CCT GCC AGC	NM_026730
Hsl(Lipe)	GAG GCC TTT GAG ATG CCA CT	AGA TGA GCC TGG CTA GCA CAG	CCA TCT CAC CTC CCT TGG CAC ACA C	NM_010719
Lpl	AAG GTC AGA GCC AAG AGA AGC A	CCA GAA AAG TGA ATC TTG ACT TGG T	CCT GAA GAC TCG CTC TCA GAT GCC CTA CA	NM_008509
Napepld	GGC CTT GGA GTC GAT TCT TCT	GTA TTT CAT AAA CCA CCT TGG TTC AT	AGG TCA AAA GGA CCA AAC CTT TTT CCA ATC TC	NM_178728
Pepck (Pck1)	GTG TCA TCC GCA AGC TGA AG	CTT TCG ATC CTG GCC ACA TC	CAA CTG TTG GCT GGC TCT CAC TGA CCC	NM_011044
Pparγ2 (Pparg)	CTA TGA GCA CTT CAC AAG AAA TTA CCA	CAC AGA GCT GAT TCC GAA GTT G	ACA CAG AGA TGC CAT TCT GGC CCA C	U09138
Scd1	ATG CTC CAA GAG ATC TCC AGT TCT	CTT CAC CTT CTC TCG TTC ATT TCC	CCA CCA CCA CCA TCA CTG CAC CTC	NM_009127

Srebp-1c (Srebf1)	GGA GCC ATG GAT TGC ACA TT	CCT GTC TCA CCC CCA GCA TA	CAG CTC ATC AAC AAC CAA GAC AGT GAC TTC C	AF286470

Additional File 1, Figure S1. (**A**) VO_2, (**B**) VCO_2 and (**C**) RER values during light and dark phases. Open symbols, $CB_1^{+/+}$ mice; closed symbols, $CB_1^{-/-}$ mice. Values are given as means ± SEM for *n*=5-7. (**D**) Gene expression levels in epididymal fat tissue of 3-week old $CB_1^{-/-}$ and $CB_1^{+/+}$ mice receiving regular chow. Open bars, $CB_1^{+/+}$ mice; closed bars, $CB_1^{-/-}$ mice. Values are given as means ± SEM for *n*=4-8.

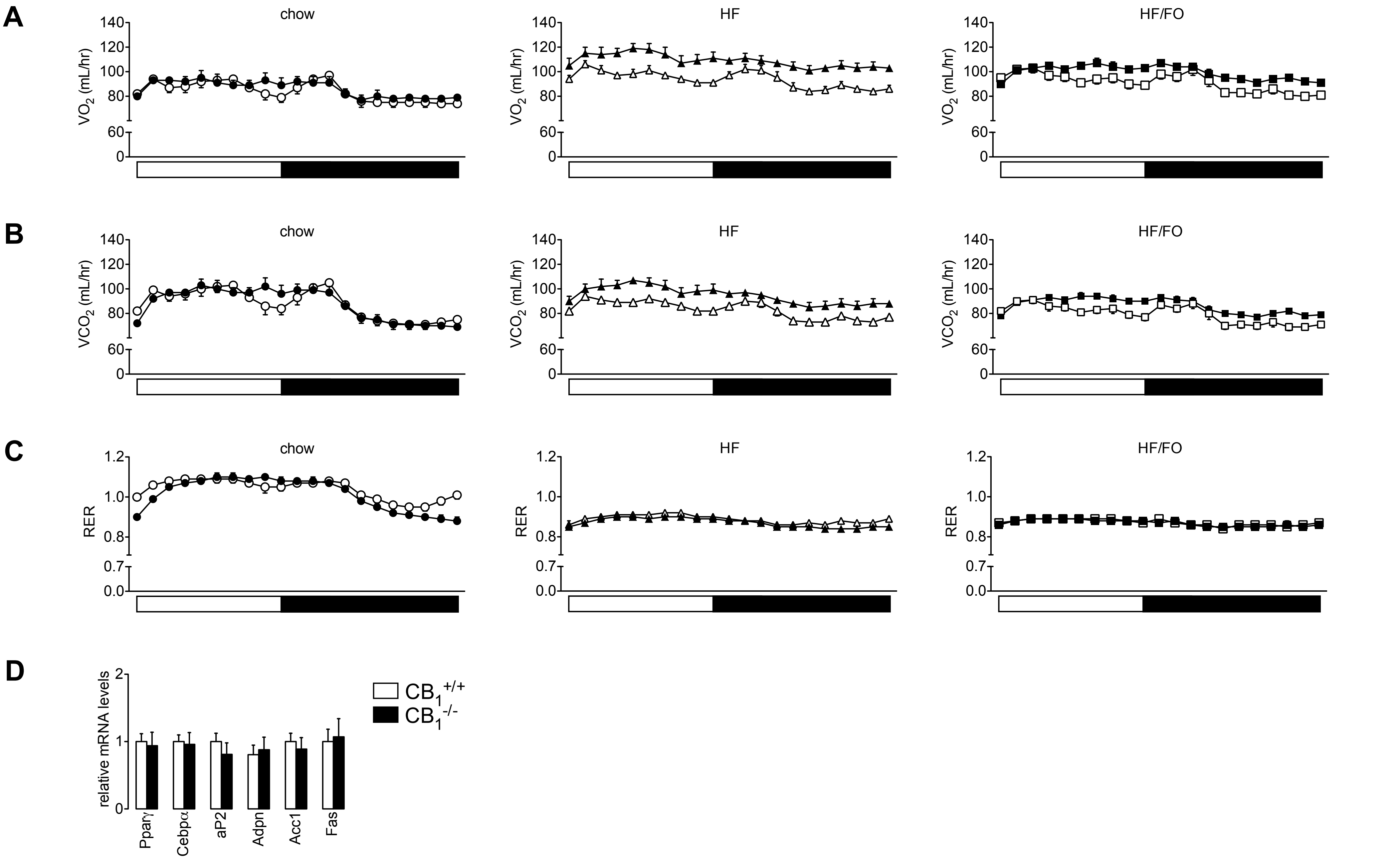

Additional File 1, Table S3. Detailed indirect calorimetry data of $CB_1^{+/+}$ and $CB_1^{-/-}$ mice fed chow, a HF or a HF/FO diet during 6 weeks.

	chow		HF		HF/FO	
	$CB_1^{+/+}$	$CB_1^{-/-}$	$CB_1^{+/+}$	$CB_1^{-/-}$	$CB_1^{+/+}$	$CB_1^{-/-}$
**Values expressed per mouse**						
Dark phase						
Carbohydrate oxidation (mg/hr)	128±7	128±6	76±2#	79±5#	68±3#	73±3#
Fat oxidation (mg/hr)	-11±2	-10±2	15±1#	19±2#	17±2#	19±2#
Energy expenditure (cal/hr)	455±16	465±20	481±9	554±23#*	470±13	506±9*
Light phase						
Carbohydrate oxidation (mg/hr)	93±6	79±3*	60±3#	57±5#	53±3#	56±4#
Fat oxidation (mg/hr)	-1±6	6±1*	17±1#	24±2#*	19±1#	22±2#
Energy expenditure (cal/hr)	397±13	401±16	436±12	509±18#*	419±16	464±10#$*
**Values expressed per gram lean body mass**						
Dark phase						
Carbohydrate oxidation (mg/hr)	25±1	28±1	15±1#	17±1#	14±1#	15±0#
Fat oxidation (mg/hr)	-2.1±0.4	-2.1±0.4	3.0±0.3#	4.2±0.4#	3.5±0.3#	3.7±0.4#
Energy expenditure (cal/hr)	89±2	101±3*	97±5	121±7#*	96±3	106±3*
Light phase						
Carbohydrate oxidation (mg/hr)	18±1	17±1	12±1#	12±1#	11±1#	12±1#
Fat oxidation (mg/hr)	-0.2±0.4	1.2±0.3*	3.5±0.3#	5.3±0.4#*	3.9±0.4#	4.6±0.5#
Energy expenditure (cal/hr)	78±1	87±3*	88±5	111±5#*	86±3	97±2#$*

Values are given as means ± SEM for n=5-7; # $p<0.05$ compared to chow group of the same genotype, \$ $p<0.05$ compared to HF group of the same genotype, * $p<0.05$ $CB_1^{-/-}$ *vs.* $CB_1^{+/+}$ (Student t-test). General linear model analysis revealed overall effects for the following parameters ($p<0.05$):

Genotype: dark phase energy expenditure per mouse, light phase energy expenditure per mouse, normalized dark phase energy expenditure, normalized light phase energy expenditure, normalized light phase fat oxidation.

Chow versus HF: dark/light phase carbohydrate/fat oxidation per mouse, light phase carbohydrate/fat oxidation per mouse, dark/light phase energy expenditure per mouse, dark/light phase normalized carbohydrate/fat oxidation, dark/light phase normalized energy expenditure.

Chow versus HF/FO: dark/light phase carbohydrate/fat oxidation per mouse, light phase carbohydrate/fat oxidation per mouse, light phase energy expenditure per mouse, dark/light phase normalized carbohydrate/fat oxidation.
